# The dawn of a new era in treating T-PLL

**DOI:** 10.18632/oncotarget.26595

**Published:** 2019-01-18

**Authors:** Alexandra Schrader, Till Braun, Marco Herling

**Affiliations:** Laboratory of Lymphocyte Signaling and Oncoproteome, Department of Internal Medicine I, Center for Integrated Oncology Köln-Bonn, University of Cologne, Cologne, Germany; Excellence Cluster for Cellular Stress Response and Aging-Associated Diseases and Center for Molecular Medicine Cologne, University of Cologne, Cologne, Germany

**Keywords:** T-PLL, ATM, HDAC inhibition, p53 reactivation, BCL2 antagonists

T-cell prolymphocytic leukemia (T-PLL) is a mature T-cell leukemia typically presenting at stages of exponentially rising lymphocyte counts in peripheral blood, accompanied by splenomegaly and bone marrow involvement [1, 2]. T-PLL is inherently highly aggressive and its notoriously refractory behavior to conventional therapeutics [3, 4] adds to its very poor prognosis. Median survival times from diagnosis are usually under 2-3 years. Up until now there is not a single FDA or EMA approved substance for T-PLL. Even after responses in 80-90% of patients to the most efficient single agent, the monoclonal antibody Alemtuzumab, relapses within 1-2 years following this treatment are the rule [2, 3]. After its market retraction for repurposing in multiple sclerosis, Alemtuzumab is currently only available in a compassionate use program. Inescapably, this has already ushered the post-Alemtuzumab era in T-PLL. Moreover, consolidating allogeneic stem cell transplantation in first best response remains the only curative option in T-PLL so far, but this procedure provides a long-term survival benefit to only 10-20% of transplanted individuals [2, 3]. With a median age at presentation of 63 years, however, only about 40% of patients with T-PLL are eligible for this intervention. Additionally, the incidence of T-PLL of ≈0.6/million in Western countries imposes major impediments to exploratory or comparative clinical trials [2–4].

The past years have witnessed a significantly improved molecular understanding of T-PLL, which culminated in a newly proposed disease model by *Schrader et al* [5]. This comprehensive work also expanded from integrated multi-level biological networks towards interrogations of molecular vulnerabilities. In conjunction with other recent seminal studies [6–8], this paper highlights the beginning of more rational-based translations of pre-clinical data into novel treatment concepts that we will likely see for T-PLL in the near future.

In *Schrader et al*, we describe that in virtually all T-PLL the genomic landscape is dominated by lesions activating the *TCL1* oncogene and by those compromising the DNA repair master regulator *ATM* [5]. This cooperative *TCL1*^up^/*ATM*^def^ genotype mainly affects key signaling branches and functions of DNA damage response (DDR) pathways, i.e. perturbations of ATM’s safeguarding tasks by TCL1 resulting in cell-death evasion. *TCL1*^up^/*ATM*^def^ jointly confer a functional signature of cellular inefficiency in alleviation of high burdens of reactive oxygen species, in maintaining telomere and genome integrity, and in activating protective p53 programs. Further alterations include oncogenes like *MYC*, epigenetic modifiers like *EZH2*, *KMTs* and *HDACs* as well as elements of micro-RNA processing (e.g. *AGO2*). We also extrapolate that overt-stage autonomous proliferation including clonal escape from niche-defined homeostatic control relies on independence from milieu input, as potentially conveyed by the highly prevalent activating *JAK/STAT* mutations or by other modes of net-activated JAK/STAT signaling [5, 6].

A pro-apoptotic response to most kinds of DNA damage is relayed through activation of p53 *via* the ATM/CHK2 axis. Explained by their hypomorphic ATM, T-PLL cells uniformly failed to generate an adequate DSB-induced p53 response [5]. Given that genetic lesions which disrupt *TP53* and its immediate regulators are infrequent in T-PLL [5], its deficient upstream activation would implicate that the functional p53 is retained at an inactive (deacetylated and MDM2-bound) state. Generally, post-transcriptional protein modifications *via* de-/acetylation (through HATs/HDACs) regulate central steps of the DDR by *(i)* direct histone modulation and by (*ii)* modifying ‘non-histone’ proteins like p53 or ATM. Consequently, we showed the efficacy of targeting such (dys)regulated acetylation *via* (H)DAC inhibitors (HDACi’s) [5]. These deductions were corroborated in unbiased *ex vivo* drug profiling studies in primary T-PLL cells [6–8]. In those screens, HDACi’s as well as p53 reactivators constituted compound classes of highest sensitivities. The combinatorial inhibitor studies by *Schrader et al* [5] finally highlighted the p53 de-repressing MDM2 inhibitor Idasanutlin to act highly efficient (also in murine T-PLL models) and in a pronounced synergism with (H)DAC inhibition. Idasanutlin reinstated repressed phospho- and acetyl-marks of p53 activity. This was enhanced by co-treatment with sub-LD50 dosages of the (H)DACi Panobinostat or the DNA-alkylator Bendamustine. Of importance, there appears to be no synthetic lethal relationship of ATM with PARP in T-PLL [5].

**Figure 1:**
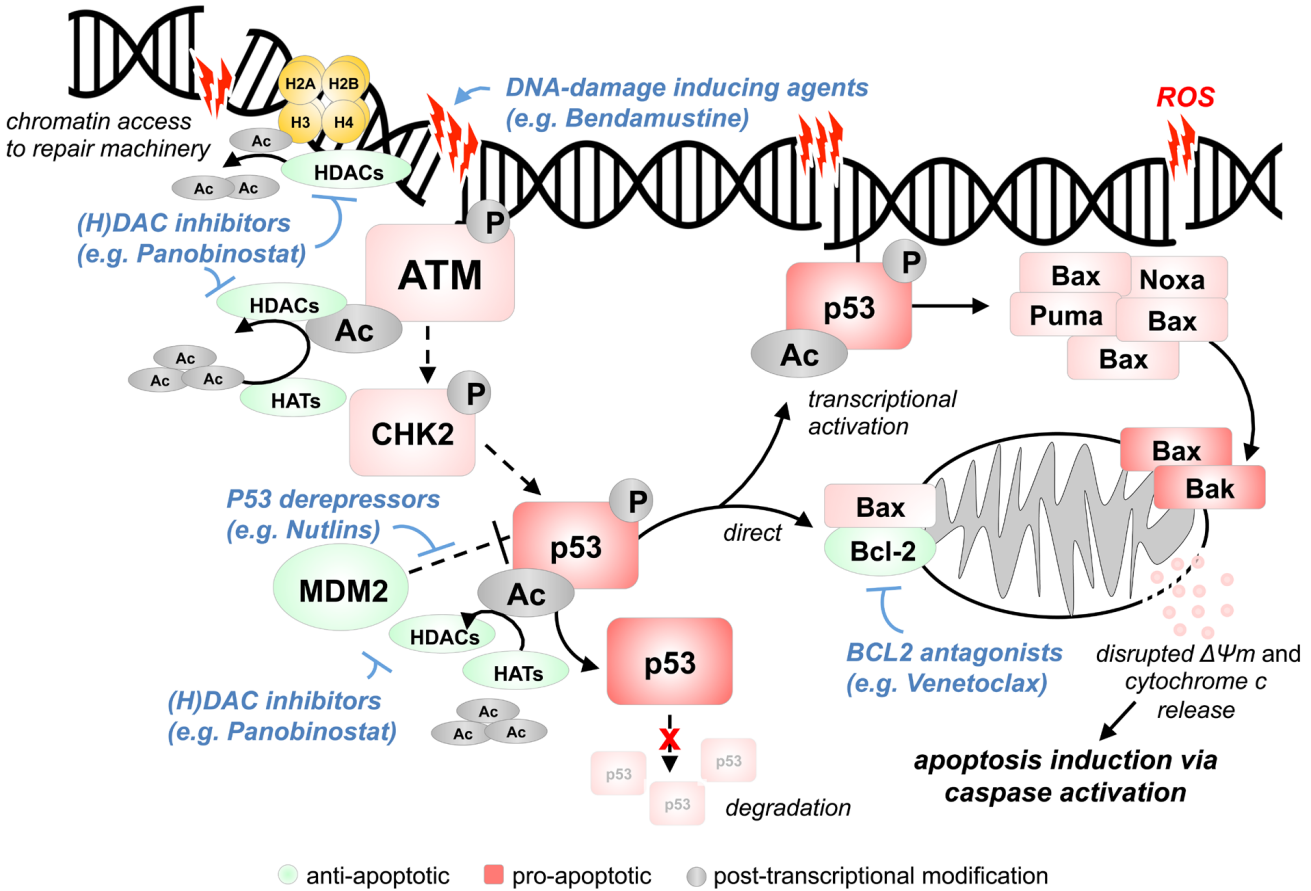
Model of enforced apoptosis induction *via* targeting of key molecular lesions in T-PLL Upon chemically/cell intrinsically (ROS) mediated DNA double strand break (DSB) induction, ATM is recruited to damage sites and undergoes auto-phosphorylation and acetylation (HAT: Tip60; HDACs: HDAC1/2). ATM kinase activation normally induces phosphorylation of downstream effectors like CHK2 and p53. Post-transcriptional modifications *via* de-/acetylation through HATs/HDACs (CBP, PCAF, hMOF and Tip60/HDAC1, SIRT1) regulate p53 activity. In T-PLL, proper activation of the otherwise intact p53 is not accomplished, most likely due to deficient ATM (deleted, mutated, modulated by TCL1). Addressing this incompetence of p53 induction and the high tonus of inactive (MDM2-bound) p53 as a central vulnerability, an enforced p53 activation through HDAC and MDM2 inhibition showed to be highly efficient in cell-death induction. Mitochondrial p53 can directly induce Bax and Bak oligomerization and antagonize the anti-apoptotic effects of Bcl-2 and Bcl-X_L_. Moreover, reactivated p53 also leads to transcriptional induction of pro-apoptotic signaling mediators like BAX, PUMA, and NOXA. Therefore, the pro-apoptotic effects of p53 reactivation could be further enhanced by Bcl-2 inhibition. The classes of (H)DAC inhibitors, MDM2 inhibitors, and Bcl-2 antagonists represent promising compounds to be interrogated for synergistic relationships, including with DNA-damage inducers.

Apoptosis induction downstream of p53 is mediated through *(i)* it´s function as a transcription factor that stimulates the expression of pro-apoptotic Bcl-2 family genes and through *(ii)* direct transcription-independent effects at the mitochondrial membrane (Figure 1). Overall, apoptosis initiation through Bcl-2 family proteins is regulated by an equilibrium of relative concentrations and affinities of pro-apoptotic BH3 proteins, anti-apoptotic Bcl-2 and Bcl-XL, and of Bax and Bak as inducers. In concordance with the described p53 incompetence of T-PLL cells and with the absence of genomic alterations in *BCL2-*family genes [5], it came to no surprise to find inhibitors targeting Bcl-2/Bcl-X_L_ to be highly active in T-PLL cells as well [6-8]. Venetoclax already showed initial promising results in first-in-man pilots [7].

Taking together, we are witnessing the exciting transition of an advanced understanding of the key molecular lesions of T-PLL towards their clinical exploitation. Within the past 2 years highly promising substance categories that specifically address the vulnerabilities of T-PLL have emerged (Figure 1). Namely, inhibitors of histone/non-histone protein deacetylation or of Bcl-2 proteins as well as p53 reactivators, and particularly combinations of those classes, will provide a new basis for future clinical trials in this chemotherapy-refractory disease.
